# Supplementary material for: An integrated technology for quantitative wide mutational scanning of human antibody Fab libraries
Source: Nat Commun. 2024 May 10;15:3974. doi: 10.1038/s41467-024-48072-z (PMC11087541; doi:10.1038/s41467-024-48072-z)
Supplement: Supplementary file 1 — Supporting Information [file 41467_2024_48072_MOESM1_ESM.pdf]

SUPPLEMENTARY INFORMATION TO

An integrated technology for quantitative wide mutational scanning of human antibody Fab libraries

Brian M. Petersen[1‡], Monica B. Kirby[1‡], Karson M. Chrispens[1], Olivia M. Irvin[1], Isabell K. Strawn[1], Cyrus M. Haas[1], Alexis M. Walker[1], Zachary T. Baumer[1], Sophia A. Ulmer[1], Edgardo Ayala[2], Emily R. Rhodes[1], Jenna J. Guthmiller[2], Paul J. Steiner[1], Timothy A. Whitehead[1, *]

[1]Department of Chemical and Biological Engineering, University of Colorado Boulder, Boulder, CO, 80305, USA

[2]Department of Immunology and Microbiology, University of Colorado Anschutz Medical Campus, Aurora, CO 80045

‡ Authors contributed equally to this work

This PDF file contains:
Supplementary Figures: S1 to S13
Supplementary Note 1: **Inferring biophysical parameters from sequencing data**

Supplementary Figures

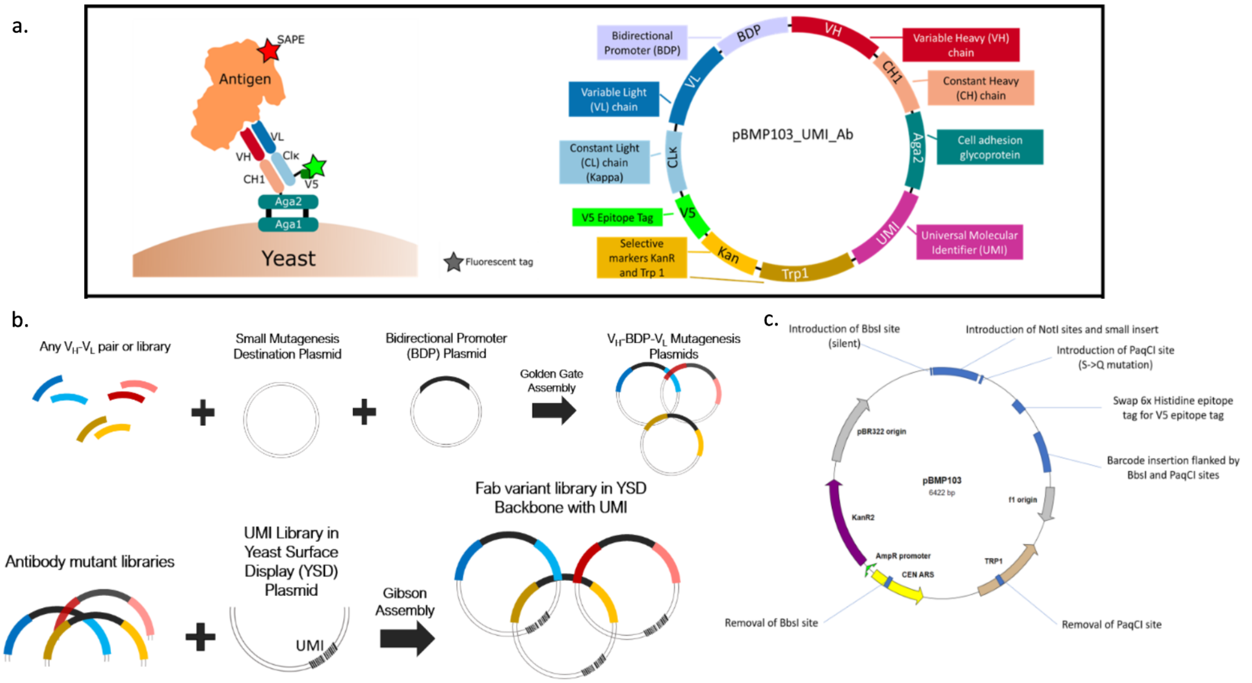

Supplementary Figure 1 | Schematic of assembly strategy with shuttle vector and yeast display mutations. a. Yeast display plasmid map highlighting most of the relevant genes (shown is kappa only; the lambda map is otherwise identical). The gene segments are not drawn to scale. **b.** Two-step cloning strategy for assembling barcoded Fab libraries. Along with a bidirectional promoter (BDP) plasmid, any V_H-V_L pair or library is assembled by Golden Gate into a minimal 3.6 kB mutagenesis plasmid containing a Cam_R selection marker, a high copy number ORI, and regions of homology to the CH1 and light chain sequence. There are small mutagenesis destination plasmids for both human kappa and lambda light chains. After mutagenesis, antibody mutant libraries are assembled by the method of Gibson with a yeast surface display plasmid containing a unique UMI. This final plasmid is identical to that in panel a. **c.** Summary of mutations in the yeast display vector. The major missense change is S5Q on CH1 necessary for encoding a PaqCI restriction site near the CDR H3 for short read sequencing pairing of UMI to the V_H gene. We also removed the light chain 6x Histidine epitope tag and replaced it with a V5 epitope tag (GKPIPNPLLGLDST) to be able to measure binding to antigens that may be His-tagged with an anti-His conjugated fluorophore. Unique PaqCI and BbsI sites are necessary for UMI-Fab pairing by short read sequencing; antibody sequences encoding these sites are not compatible with the short read sequencing protocol.

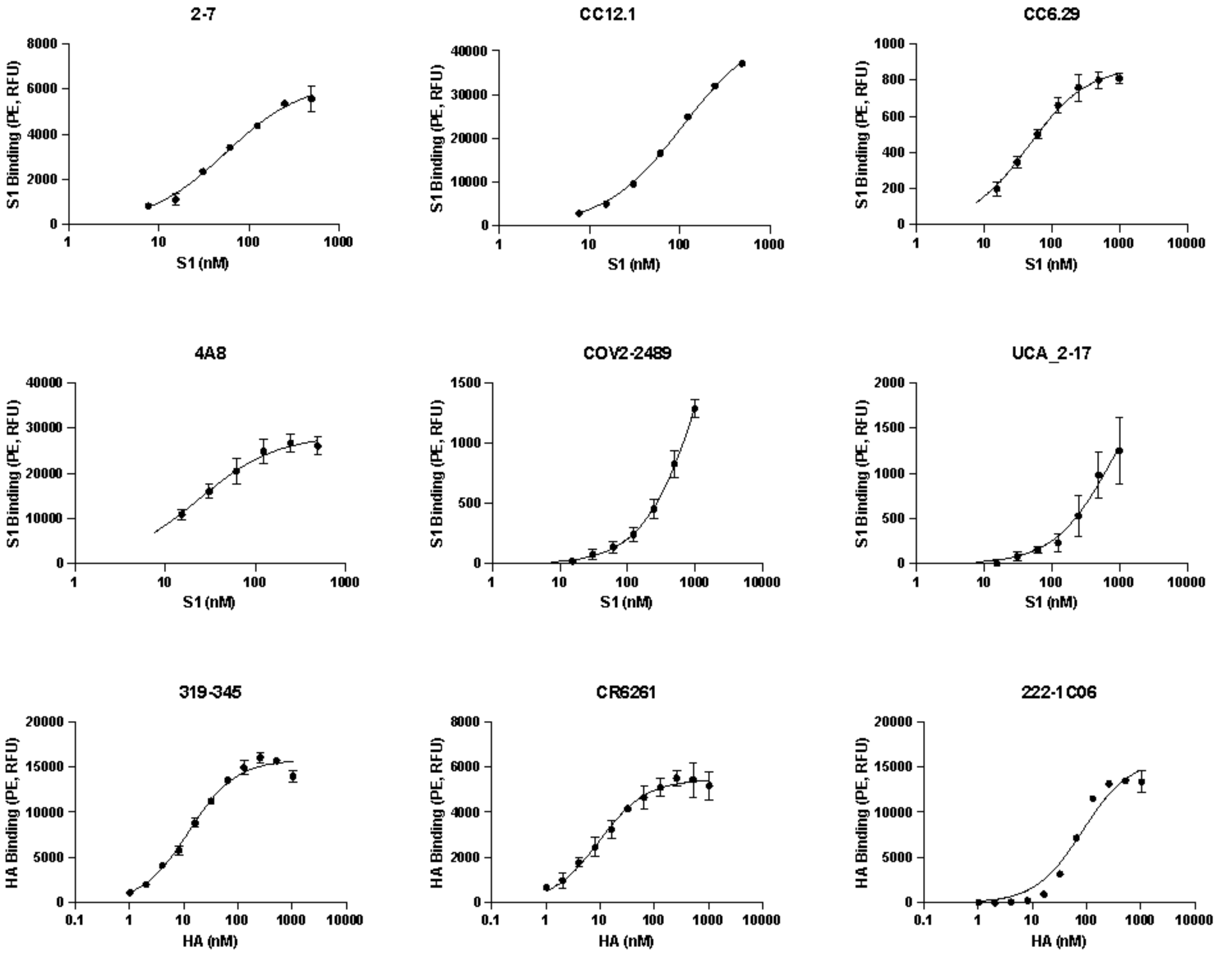

Supplementary Figure 2 | Yeast surface display titrations. Isogenic yeast surface titrations for antibodies reported in main text Figure 1c. Error bars represent 1 s.d. of 2 measurements. The curve fit shown is a Hill equation where the Hill coefficient is constrained to unity.

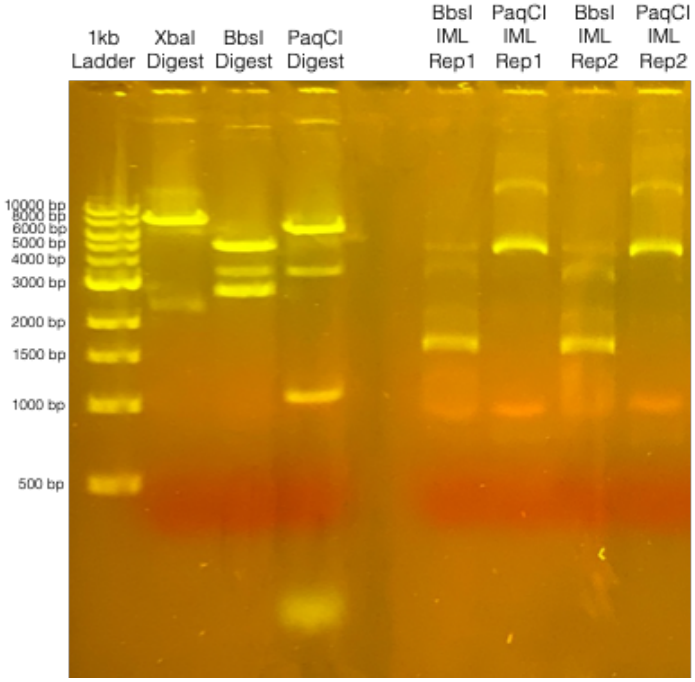

Supplementary Figure 3 | Intramolecular Ligation Products. Intramolecular ligation (IML) reaction products from 1 µg of barcoded 4A8/CC12.1/COV2-2489 Fab library (lanes 6-9) were separated by a 1% (w/v) agarose gel. Lanes 2-4 show individual controls reactions without ligase. The ligation product from BbsI is 1.8 kB, while that from PaqCI is 6.4 kB. In these reactions, the UMI is paired to the V_L with BbsI intramolecular ligation and to the V_H with PaqCI. Biological replicates (Rep1, Rep2) were performed, yielding reproducible band sizes and intensities. The gel shown is the unmodified and uncropped image.

			True Frequencies of Correct Pairing			P values (T test)						
Polymerase	PCR Cycles	PCR Clean-up Method	Replicate 1	Replicate 2	Replicate 3	Q5, 25x Cycles, rSAP	Phusion, 12x, rSAP	KAPA, 14x, rSAP	Q5, 14x, rSAP	Q5, 14x, Column	KAPA, 14x, Column	Phusion, 12x, Column
Q5	25	rSAP	60%	59%	55%	5.00E-01						
Phusion	12	rSAP	90%	92%	91%	1.24E-05	5.00E-01					
KAPA	14	rSAP	89%	87%	86%	3.34E-05	8.99E-03	5.00E-01				
Q5	14	rSAP	82%	83%	81%	6.68E-05	3.29E-04	7.13E-03	5.00E-01			
Q5	14	Column	85%	78%	79%	5.43E-04	5.16E-03	2.57E-02	2.75E-01	5.00E-01		
KAPA	14	Column	86%	89%	92%	6.74E-05	1.29E-01	2.13E-01	1.00E-02	1.98E-02	5.00E-01	
Phusion	12	Column	82%	84%	78%	3.10E-04	3.75E-03	2.38E-02	3.53E-01	4.09E-01	1.90E-02	5.00E-01

			True Frequencies of Correct Pairing			P values (T test)	
Polymerase	PCR Cycles	PCR Clean-up Method	Replicate 1	Replicate 2	Replicate 3	Q5, 25x Cycles, rSAP	IML Control, 25x Cycles, rSAP
Q5	25	rSAP	60%	59%	55%	5.00E-01	
IML Control Q5	25	rSAP	64%	73%	65%	2.10E-02	5.00E-01

Supplementary Figure 4 | Optimization of PCR amplicon preparation for barcode-Fab haplotyping. Three isogenic plasmids (one CC12.1 variant, two 4A8 variants) were mixed at different molar ratios and the intramolecular ligation for the V_L chain and amplicon protocols were performed in triplicate (n=3). We varied the following parameters: polymerase, number of PCR cycles, and PCR clean-up method (rSAP (New England Biolabs) or column cleanup). Amplicons were deep sequenced on the same MiSeq flow cell, and observed frequencies of pairing were extracted. True frequencies of correct pairing ($freq_{true}$) were obtained for the lowest abundant variant using the following equation:

$$freq_{true} = \frac{freq_{obs} - f}{1 - f} \quad (22)$$

Here, $freq_{obs}$ is the observed frequency by deep sequencing and f is the fraction of the lowest abundant variant. As this fraction goes to zero the true frequency is identical to the observed frequency. True frequencies and p-values from paired, one-tailed t-tests are reported. We also report true frequencies and p-values of performing the intramolecular ligation separately on individual plasmids (IML control). The protocol chosen for barcode-Fab haplotyping is highlighted in green.

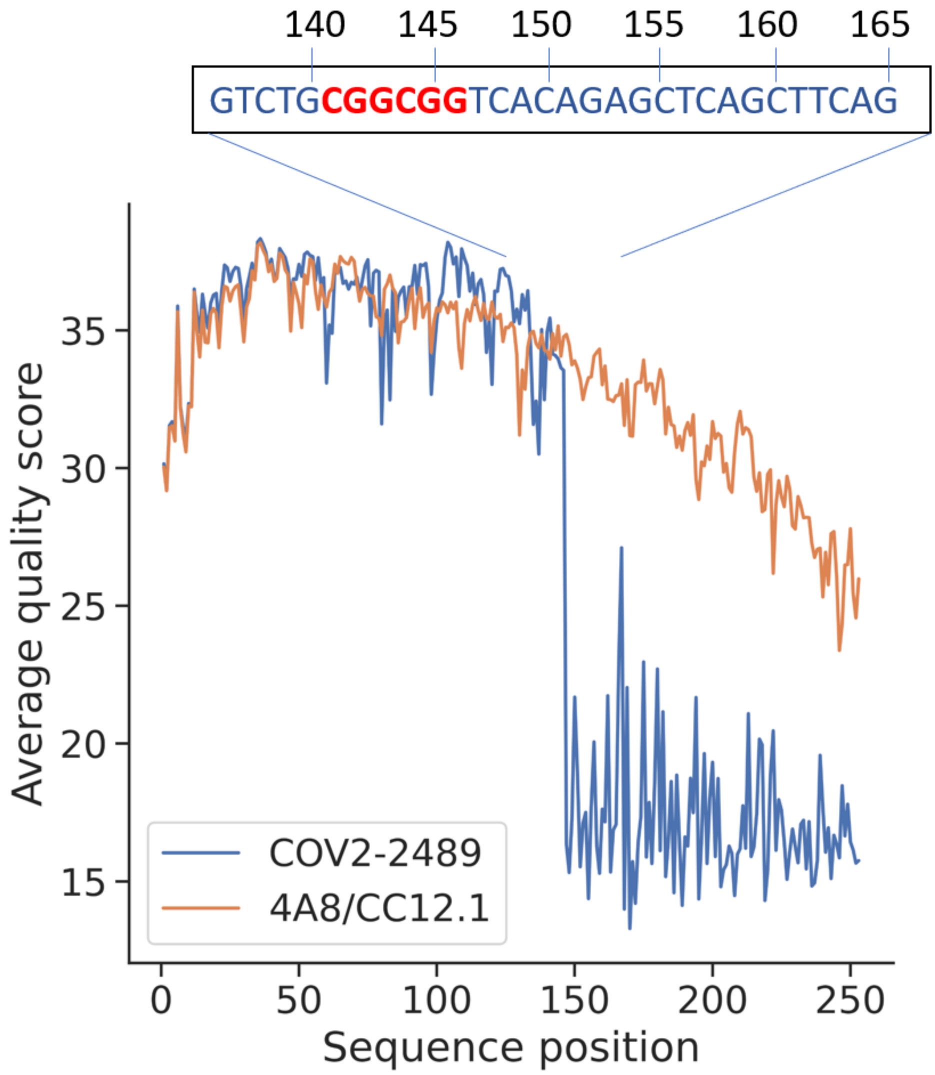

Supplementary Figure 5 | Repeated 'CGGCGG' motif in COV2-2489 sequences causes drop in quality at nucleotide 147 on V_H reverse read. Average quality score vs. sequence position for 4A8 & CC12.1 antibodies (orange) compared to COV2-2489 (blue). The inset shows the nucleotide sequence adjacent to the drop in quality score.

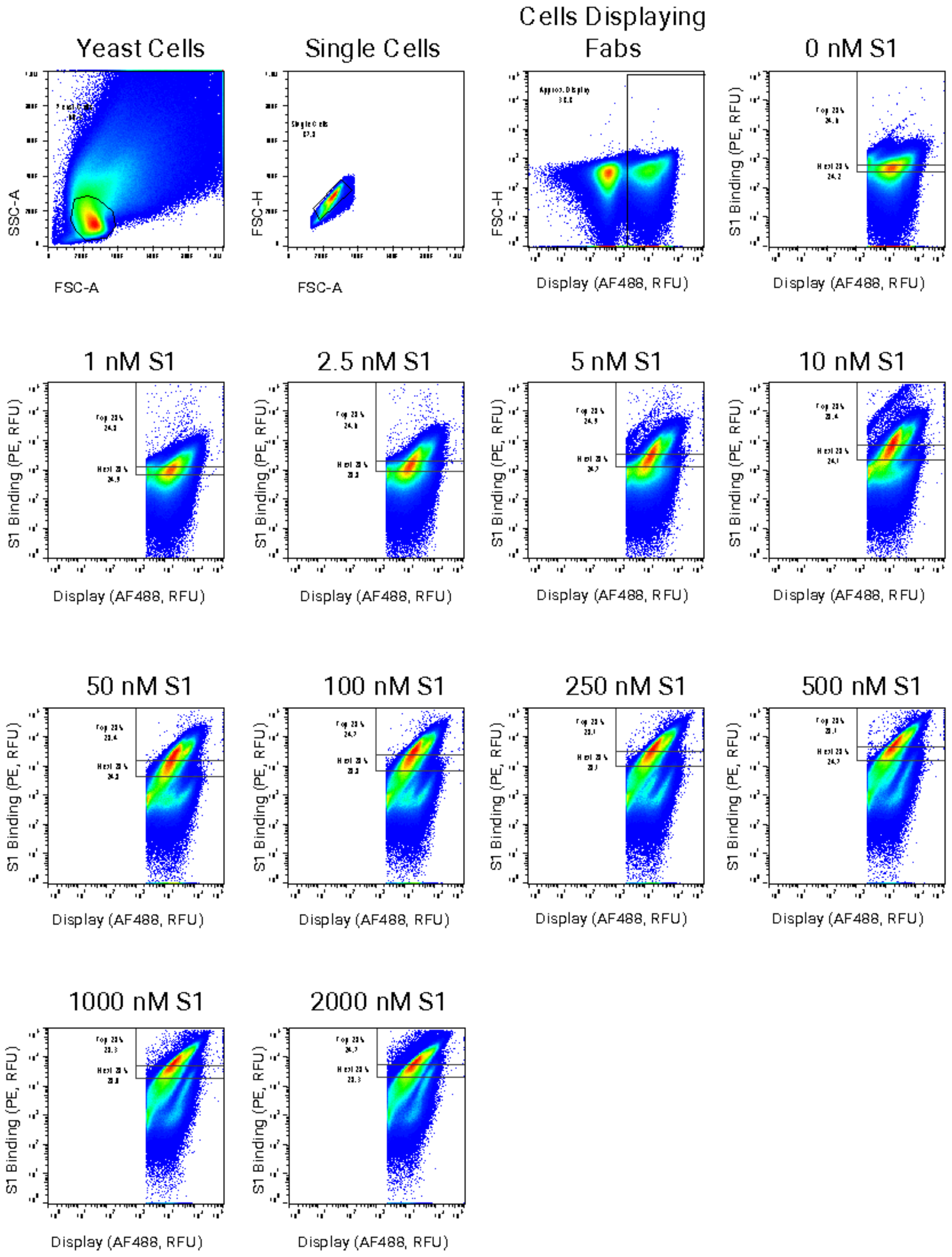

Supplementary Figure 6 | Cytograms from first sort with S1 and 4A8/CC12.1/COV2-2489 Antibodies. Cytograms showing sorting gates for first demonstration of the method with mixed Abs against S1. Cells were first gated for yeast cells, single cells, and cells displaying Fabs before being gated and sorted for the top 25% and next 25% bins.

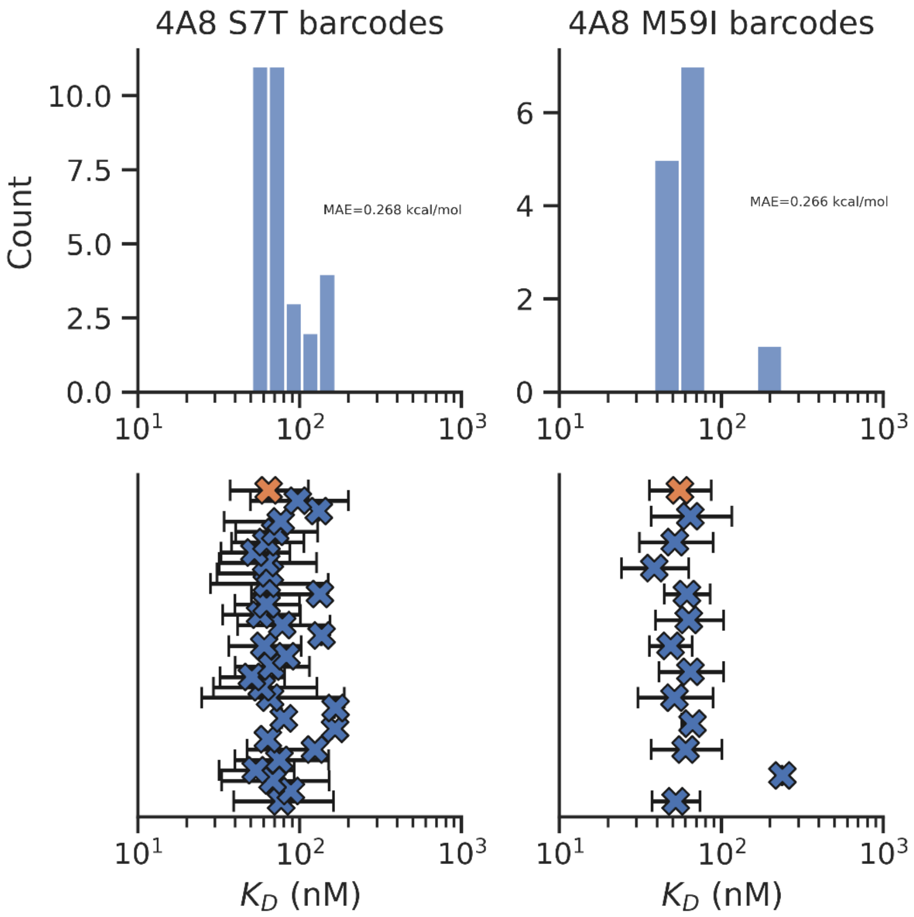

Supplementary Figure 7 | MLE K_D estimation for grouped barcodes with 95% confidence intervals. *(Top row) Histograms of MLE K_D estimates for each barcode with calculated mean absolute error from isogenic titration data. (Bottom row) 95% confidence intervals for each barcode (blue X, S7T: n=30, M59I: n=12) and from barcodes collapsed by variant (orange X)*

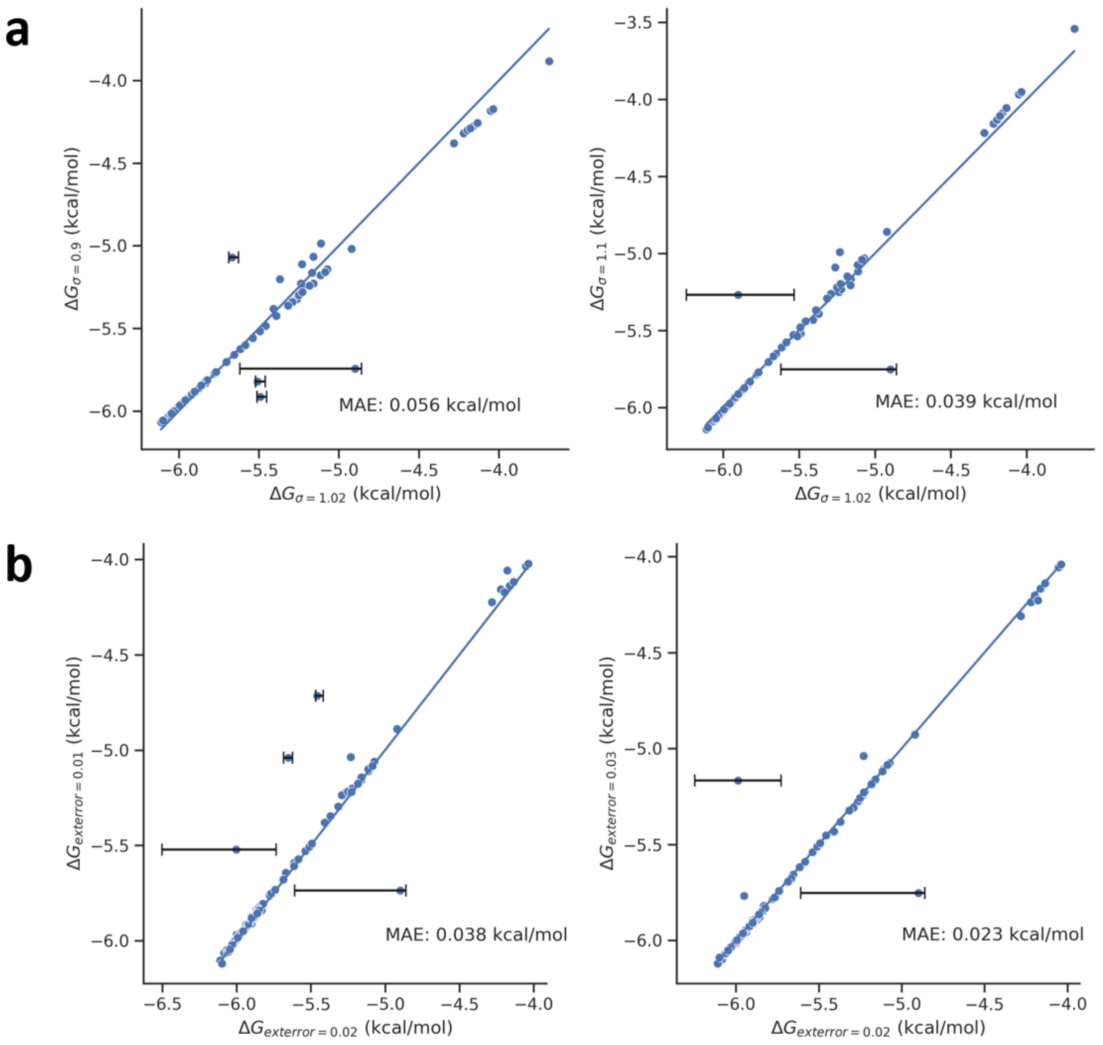

Supplementary Figure 8 | MLE sensitivity analysis of global parameters. *MLE calculated $\Delta G_{binding}$ values, relative to a 1 mM reference state, for 4A8 and CC12.1 data with different values of σ, the width of the isogenic lognormal distribution (Equation (2)) (a) and extrinsic error (Equation (15)) (b) with 95% confidence intervals for outliers (outliers defined as >0.3 kcal/mol MAE). Data were filtered to remove low counts and non-converged values.*

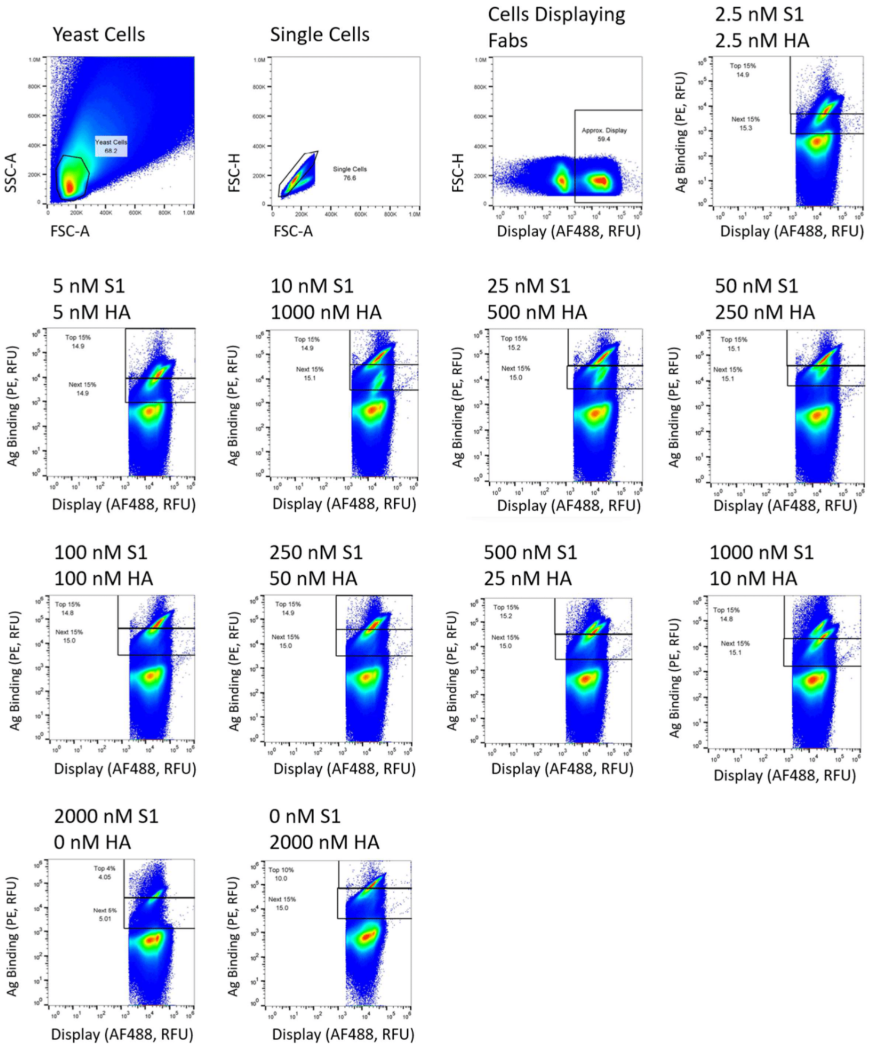

Supplementary Figure 9 | Cytograms from YL008 mixed antigen sort with S1 and HA. *Cytograms showing sorting gates for mixed antibody, mixed antigen sort.*

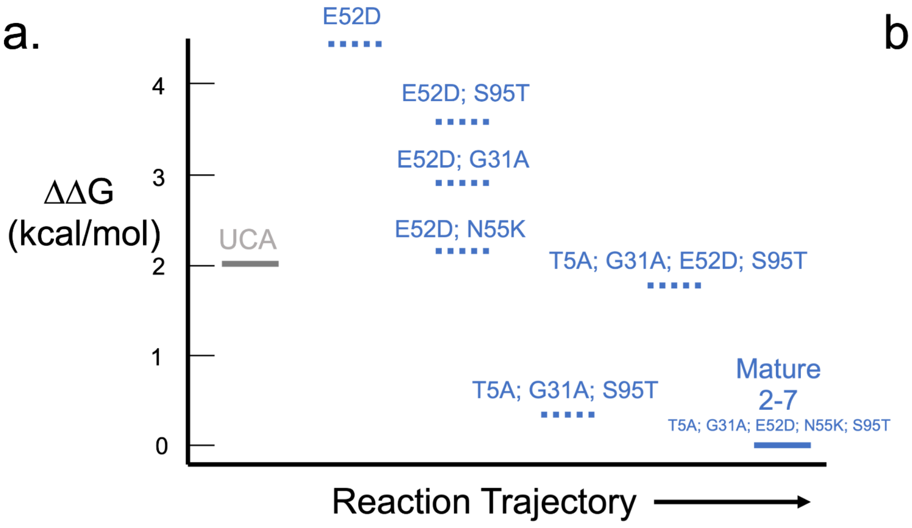

b.

MUTATION	ΔΔG (kcal/mol)
A5T	0
A31G	1.9
D52E	-1.6
K55N	1.4
T95S	0.4

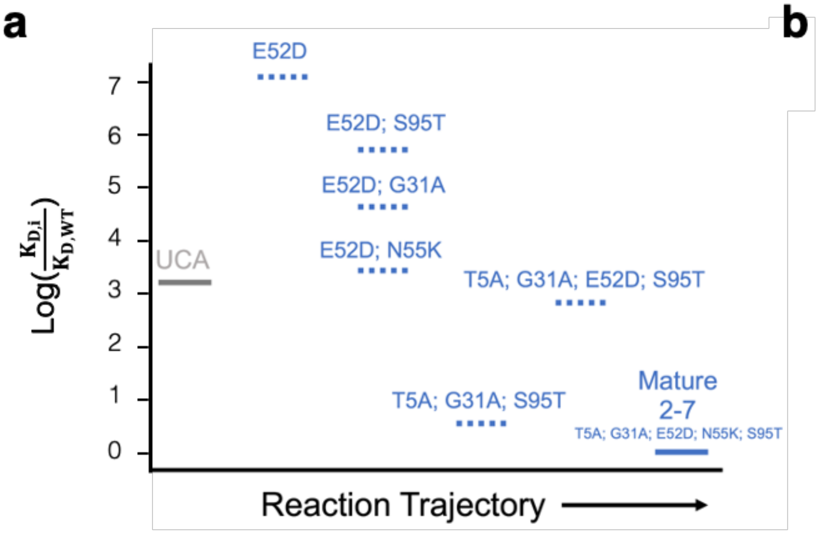

b

Mutation	$\mathrm{Log}(\frac{K_{D,i}}{K_{D,WT}})$
A5T	0.0
A31G	3.1
D52E	-2.6
K55N	2.3
T95S	0.6

Supplementary Figure 10 | Potential development trajectory for SARS-CoV-2 antibody 2-7. (a) Sampling of 6 of the 30 potential intermediates between the UCA and mature 2-7. The affinity of each variant is shown as ΔΔG (kcal/mol) relative to the mature 2-7 antibody. Mature 2-7 has an inferred K_d of 9.6 nM and the UCA a K_d of 255 nM. (b) LASSO regression one body weights for ΔΔG for the five V_L mutations. Weights in kcal/mol are shown relative to the mature 2-7 Ab. The E52D mutation is energetically unfavorable and unlikely to have appeared except in conjunction with the N55K mutation.

SUPPLEMENTARY NOTE 1:

1. INFERRING ANTIBODY-ANTIGEN BIOPHYSICAL PARAMETERS FROM SEQUENCING DATA: EXECUTIVE SUMMARY

In deep mutational scanning, a population of mutational variants of a protein is passed through a selection or screen; this screen changes the underlying frequencies of each of these variants. Deep sequencing is used to count each variant in the population, which is used to infer the frequency of each variant in the population in a reference population and after the screen. This frequency change is converted to some score that, ideally, relates to the functional properties of the variant. This technical note describes our framework for inferring, from this processed sequence data, both dissociation constants and maximum fluorescence for antibody variants encoded in yeast displayed protein libraries screened by fluorescence activated cell sorting (FACS).

In FACS, populations are screened by collecting cells with fluorescence above a certain gating fluorescent threshold, or between two fluorescent gating thresholds; cells sorted according to these fluorescent gates are said to be sorted into bins. A clonal population of cells will exhibit a mean fluorescence with a certain variance according to cell size, surface density of displayed proteins, or other factors. Thus, only a fraction of cells for each variant will exceed the fluorescence threshold needed for collection into a given bin. Furthermore, if the fraction of cells that are sorted into a bin is known, one can infer the likely mean fluorescence for a given variant at that labeling concentration. Finally, as described in further detail below, sequencing data and other experimental observables can be used to infer the fraction of cells collected by the gating strategy and thus the mean fluorescence of a variant for a given labeling concentration. Some of the descriptions below come in part from Kowalsky et al[1], and Kowalsky et al[2].

We seek to infer variant-specific dissociation constants ($K_{d,i}$) using, for example, the Hill equation below:

$$\underline{F_i} = \left(F_{max,i} - F_{min}\right)\frac{[L]}{K_{d,i}+[L]} + F_{min} \qquad (1)$$

Here is the mean fluorescence of cells displaying variant i at a given labeling concentration [L], $F_{max,i}$ is the maximum fluorescence for the variant i, and F_{min} is cellular autofluorescence.

Intuitively, if we can infer the mean fluorescence at different labeling concentrations ([L]), we can reconstruct isothermal titrations for each variant i (e.g. $\underline{F_i}$ vs. [L]) to find a best fit $K_{d,i}$ and $F_{max,i}$ using non-linear regression. An example from barcode ATGCACACATTTAAAGCTGT corresponding to variant 4A8 M59I is shown below in **Fig Note S1**.

We can approach this inference problem by regression, as it allows for the quality of the model fit to the data using the chi squared metric while also giving robust methods for confidence interval testing. As will be shown, we can also use maximum likelihood estimation in a quantitatively identical way. However, we cannot regress on the reconstructed mean fluorescence, as error is not distributed uniformly in both directions. Instead, we regress on the vector of *probabilities* of sorting into each bin.

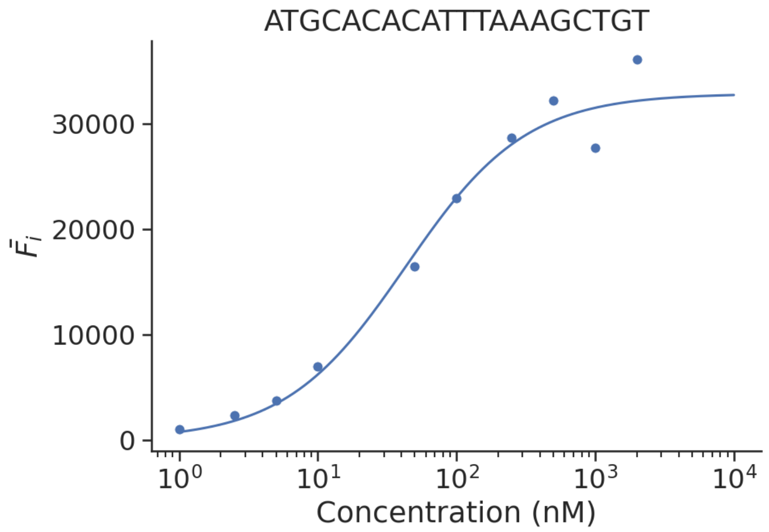

Supplementary Figure 11: *Fluorescence reconstruction for barcode ATGCACACATTTAAAGCTGT at 10 labeling concentrations.*

In summary, we label our population of antibody variants at different antigen concentrations and use FACS to sort these antibody variants into different bins. Sequencing of these populations allows us to reconstruct the likely mean fluorescence for a given labeling concentration by inferring the fraction of each variant that is present in a binned population. Summing over all labeling concentrations allows us to find the most likely parameter value for dissociation constants, the confidence interval associated with that parameter estimation, and the quality of the fit using weighted nonlinear regression.

2. WHAT IS THE PROBABILITY OF A CELL COLLECTED ABOVE A CERTAIN FLUORESCENT THRESHOLD?

Let's call this fluorescence threshold a gating fluorescence (F_g) and ask for the probability that a given clone i exhibiting a mean fluorescence intensity ($\underline{F_i}$) will be captured by this gate. A graph of this relationship is below:

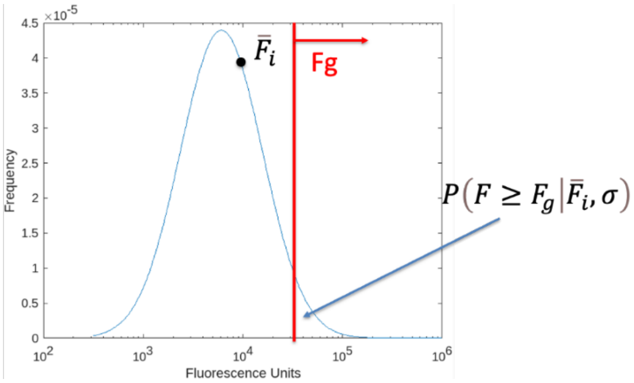

Supplementary Figure 12: *A theoretical frequency histogram for yeast cells isogenically expressing a clone i.*

Since fluorescence measurements of clonal population of displaying cells are log-normally distributed in flow cytometry[3,4], the probability can be calculated by regular statistical calculations:

$$P(\underline{F}_i,\sigma) = \frac{1}{2} - \frac{1}{2} erf\left(\frac{lnF_g - ln\underline{F}_i + \frac{1}{2}\sigma^2}{\sigma\sqrt{2}}\right) \tag{2}$$

The other variable, σ, represents the natural log of the standard deviation of the log-normal distribution from a clonal population of cells. 'erf' is the error function used to numerically integrate a Gaussian probability distribution. Equation (2) is the fundamental equation that allows us to apply statistical calculations to derive dissociation constants.

3. WE CAN FIND THE PROBABILITY OF A CELL COLLECTED BETWEEN TWO FLUORESCENT THRESHOLDS.

Assume we have a square gate set up with the lower bound some F_{g2} and the upper bound F_g. Keeping the same definitions as above, we can rewrite a similar equation as (2) for the probability above F_{g2}:

$$P(\underline{F}_i,\sigma) = \frac{1}{2} - \frac{1}{2} erf\left(\frac{lnF_{g2} - ln\underline{F}_i + \frac{1}{2}\sigma^2}{\sigma\sqrt{2}}\right) \tag{3}$$

Writing the probability of that cell landing between the two gates becomes:

$$P(\underline{F}_i,\sigma) = P(\underline{F}_i,\sigma) - P(\underline{F}_i,\sigma) \tag{4}$$

$$P(\underline{F}_i,\sigma) = \frac{1}{2} erf\left(\frac{lnF_g - ln\underline{F}_i + \frac{1}{2}\sigma^2}{\sigma\sqrt{2}}\right) - \frac{1}{2} erf\left(\frac{lnF_{g2} - ln\underline{F}_i + \frac{1}{2}\sigma^2}{\sigma\sqrt{2}}\right) \tag{5}$$

Thus, the probability p_{ijk} that a given cell displaying variant i can be captured in bin j at labeling concentration k is given by the following expression:

$$p_{ijk} = P(\underline{F_i}, \sigma) = \frac{1}{2} erf\left(\frac{lnF_{gjk} - ln\underline{F_i} + \frac{1}{2}\sigma^2}{\sigma\sqrt{2}}\right) - \frac{1}{2} erf\left(\frac{lnF_{g2jk} - ln\underline{F_i} + \frac{1}{2}\sigma^2}{\sigma\sqrt{2}}\right) \quad (6)$$

Note that if we have two bins with a shared boundary, we can write the *sum of the two probability distributions* as:

$$p_{ijk} + p_{ij+1k} = P(\underline{F_i}, \sigma) + P(\underline{F_i}, \sigma) = \frac{1}{2} - \frac{1}{2} erf\left(\frac{lnF_{g2} - ln\underline{F_i} + \frac{1}{2}\sigma^2}{\sigma\sqrt{2}}\right) \quad (7)$$

4. WHAT DO THESE PROBABILITIES LOOK LIKE IN PRACTICE?

For a typical monovalent binding experiment, one labels yeast cells displaying a binding protein with a fluorescently conjugated ligand. We have found that σ for phycoerythrin (SAPE) labeled populations range from 0.9-1.05 [2]. Let's assume a $\sigma = 1.02$ for this example. We find that many protein-ligand interactions we consider in lab to be well fit by a Hill equation with no cooperativity:

$$\underline{F_i} = (F_{max,i} - F_{min}) \frac{[L]_o}{K_{d,i} + [L]_o} + F_{min} \quad (1)$$

For the phycoerythrin (SAPE) labeled populations we usually consider, a typical value of F_{min} representing cell autofluorescence is 350 MFI in our experimental set-up using a Sony SH800 cell sorted with a 488 nm laser and compensation for fluorescein. The two protein-specific terms are the max fluorescence ($F_{max,i}$) and the dissociation constant $K_{d,i}$ for the interaction. These will be variant-specific. For reasonably expressed and well-behaved proteins our $F_{max,i}$ is typically in the 50,000 MFI range. Let's nondimensionalize the ligand concentration so we can remove one variable.

$$\underline{F_i} = (F_{max,i} - F_{min}) \frac{\frac{[L]}{K_d}}{1 + \frac{[L]}{K_d}} + F_{min} \quad (8)$$

Supplementary Table 1 reports the resulting probability lookup table:

$\frac{[L]}{K_d}$	$\underline{F_i}$	p_{ijk} (Fg = 2000)	p_{ijk} (Fg = 5000)	p_{ijk} (Fg = 10000)	p_{ijk} (Fg = 25000)
0	350	.01	<.001	<.001	<.001
0.1	4860	.64	.30	.11	.02
0.2	8625	.82	.51	.26	.06
0.3	11800	.89	.63	.36	.11
0.4	14500	.92	.70	.44	.15
0.5	16900	.94	.75	.50	.19
1	25200	.97	.86	.65	.31
5	41725	>.99	.94	.81	.50

This table shows that the large standard deviation resulting from square gating gives useful probabilities at many different gating fluorescence values representing different dissociation constants and/or max fluorescence values.

5. WE CAN INFER THE PROBABILITY USING FREQUENCY DATA

Our observable for deep sequencing experiments is a set of read counts for variant **i** in each bin **j** and for each labeling concentration **k** (let's call these read counts r_{ijk}). Additionally, we have the **r**eference read counts we can observe for variant **i** (let's call this r_{ir}). We can directly convert observables to probabilities of sorting into a given bin j by comparing these read counts to those from the reference population. The reference population is critically important given that the comparison is the probability of being captured by a gate relative to the condition of no gate. Therefore, your reference population must be identical except for the fluorescence gate you sort at.

We write the probability as the number of cells of variant i collected in the jth bin and kth labeling concentration (x_{ijk}) relative to the number of cells of variant i in the reference population (x_{ir}):

$$p_{ijk} = \frac{x_{ijk}}{x_{ir}} \tag{9}$$

The frequency of variant i (f_{ijk}, f_{ir}) is just the number of counts observed divided by all counts, so we can write:

$$p_{ijk} = \emptyset \frac{\frac{r_{ijk}}{\sum_i r_{ijk}}}{\frac{r_{ir}}{\sum_i r_{ir}}} \tag{10}$$

Here $\emptyset$ is the total fraction of cells collected in the sorting bin relative to the reference population, and the frequency of each variant has been converted to experimental observables derived from deep sequencing. Equation (10) is the second fundamental equation because it states that the probability p_{ijk} (set by $F_{max,i}$ and $K_{d,i}$) we observe for a given labeling concentration k and bin j are a function of the observables from the deep sequencing experiment.

6. SOURCES OF NOISE IN RECONSTRUCTING FLUORESCENCE FROM EXPERIMENTAL OBSERVABLES

A major challenge for sequence-function reconstruction experiments comes from determining the appropriate confidence level set for each experimental measurement. This is important as low and high values of p_{ijk} give large uncertainties in the measurement of $\underline{F}_{ik}$ (see **Fig Note S3** below).

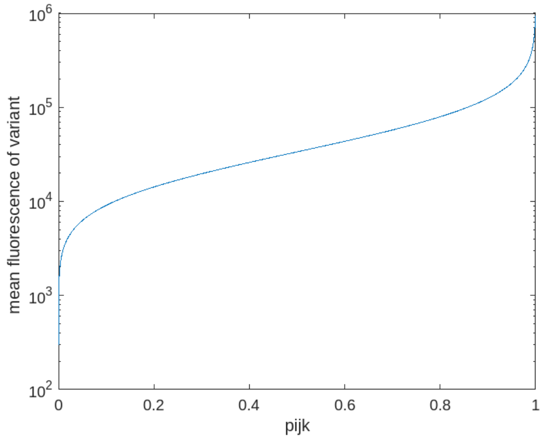

Supplementary Figure 13: *Mean fluorescence of variant as a function of* p_{ijk} *- the probability of sorting into a bin above some* F_g *= 30,000 MFI. Low and high observed probabilities result in large, one-tailed uncertainties in the value of the mean fluorescence.*

For parameter inference it is important to identify and quantify sources of noise in the fluorescence reconstruction. Intrinsic noise comes in the act of sorting discrete cells, preparing amplicons from yeast cells by PCR, and sequencing discrete nucleic sequences. Extrinsic noise results from the efficiency of the overall process of cell sorting and recovery.

We have previously shown[5] that deep sequencing read counts from FACS data can be evaluated according to Poisson probability distributions. We have previously determined the propagation of errors for Poisson noise in the counts of the reference and selected populations[6]. Although this is an underestimate of error because population bottlenecks occur during cell sorting, outgrowth, and amplicon prep leading to overdispersion, we find empirically that Poisson noise is a reasonable approximation for well-designed experiments. By propagation of errors, we can determine the variance associated with the value of p_{ijk}:

$$\sigma^2_{pijk,intrinsic} = \sigma^2_{rijk}\frac{\partial pijk}{\partial rijk}^2 + \sigma^2_{rir}\frac{\partial pijk}{\partial rir}^2 \qquad (11)$$

We can approximate the Poisson noise as

$$\sigma^2_{rijk} = r_{ijk};\ \sigma^2_{rir} = r_{ir} \qquad (12)$$

$$\sigma^2_{pijk,intrinsic} = r_{ijk}\left(\emptyset\frac{\frac{1}{\sum_i r_{ijk}}}{\frac{r_{ir}}{\sum_i r_{ir}}}\right)^2 + x_{ir}\left(-\emptyset\frac{\frac{r_{ijk}}{\sum_i r_{ijk}}}{\frac{r_{ir}^2}{\sum_i r_{ir}}}\right)^2 \qquad (13)$$

$$\sigma^2_{pijk,intrinsic} = \frac{p_{ijk}{}^2}{r_{ijk}} + \frac{p_{ijk}{}^2}{r_{ir}} \qquad (14)$$

The other source of error is extrinsic relating to error rate in the sorting itself – what is the probability of a mis-sorting event? This appears to be 2% in our experimental set-up, but we expect that this error rate may vary.

$$\sigma^2_{pijk,extrinsic} = (0.02)^2 \quad (15)$$

In the experiments presented in Figures 4 and 5 of the main text, we included Fab nonbinders to measure this error directly from the sequencing data. For these experiments, these values were observed to be 0.014, close to the values used in the initial experiment.

Taken together by propagation of error, we end up with the following result for the uncertainty associated with probability:

$$\sigma_{pijk} = \sqrt{\sigma^2_{pijk,extrinsic} + p_{ijk}{}^2\left(\frac{1}{r_{ijk}} + \frac{1}{r_{ir}}\right)} \quad (16)$$

7. PARAMETER ESTIMATION USING MAXIMUM LIKELIHOOD ESTIMATION

The log likelihood framework states that the parameter set most likely to fit a given set of data occurs with maximization of the summation of the log probabilities of each experimental measurement:

$$LL_i(K_{d,i}, F_{max,i}) = \left(\sum_{jk} logP\left(Model_{ijk}\right)\right) \quad (17)$$

Here, $Model_{ijk}$ is the model probability (given parameters $K_{d,i}, F_{max,i}$) of a variant i being sorted into bin j at labeling concentration k. We must assume some probability distribution – given the sources of noise and the fact that reference and sorted counts are typically >10, a Gaussian probability distribution is justifiable here. Expanding terms, we can write:

$$LL_i(K_{d,i}, F_{max,i}) = \left(\sum_{jk} logGaussianPDF\left(Model_{ijk}\right)\right) \quad (18)$$

Expanding the Gaussian probability distribution and removing constant terms, we arrive at:

$$LL_i(K_{d,i}, F_{max,i}) = \sum_{jk} -\frac{1}{2}\left(\frac{p_{ijk} - Model_{ijk}}{\sigma_{ijk}}\right)^2 \quad (19)$$

Note that maximizing this expression is equivalent to minimizing the weighted sum of square errors or the chi squared metric. The algorithm changes the probabilities of $Model_{ijk}$ by changing parameters in the Hill function, and we use off-the-shelf optimization software to find the minimization of the function.

$$-LL_i(K_{d,i}, F_{max,i}) = \sum_{jk}\left(\frac{p_{ijk} - Model_{ijk}}{\sigma_{ijk}}\right)^2 \quad (20)$$

8. CONFIDENCE INTERVALS USING MAXIMUM LIKELIHOOD ESTIMATION

Using a MLE framework that minimizes the chi squared metric (χ^2_{min}) results in the simplification of confidence interval measurements. As such, we follow standard approaches[7,8] for determining 95% confidence intervals using the critical value of the F distribution statistic ($F_{0.05}$) using the following equation:

$$\frac{\chi^2}{\chi^2_{min}} \leq \frac{n-2}{n-1}\left(1 + \frac{n}{n-1} F_{0.05}\,(n-1, n)\right) \tag{21}$$

Where n is the number of experimental data points (here, the number of bins used for MLE), and χ^2 is the chi squared metric for given parameter values of K_{di} and F_{maxi}.

Supplementary References

1. Kowalsky, C. A. *et al.* High-resolution sequence-function mapping of full-length proteins. *PLoS One* **10**, 1–23 (2015).
2. Kowalsky, C. A. *et al.* Rapid fine conformational epitope mapping using comprehensive mutagenesis and deep sequencing. *J. Biol. Chem.* **290**, 26457–26470 (2015).
3. Cossarizza, A. *et al.* Guidelines for the use of flow cytometry and cell sorting in immunological studies (second edition). *Eur. J. Immunol.* **49**, 1457–1973 (2019).
4. Boder, E. T. & Dane Wittrup, K. Optimal screening of surface-displayed polypeptide libraries. *Biotechnol. Prog.* **14**, 55–62 (1998).
5. Whitehead, T. A. *et al.* Optimization of affinity, specificity and function of designed influenza inhibitors using deep sequencing. *Nat. Biotechnol.* **30**, 543–548 (2012).
6. Klesmith, J. R., Bacik, J. P., Michalczyk, R. & Whitehead, T. A. Comprehensive Sequence-Flux Mapping of a Levoglucosan Utilization Pathway in E. coli. *ACS Synth. Biol.* **4**, 1235–1243 (2015).
7. Wittrup, K. D., Tidor, B., Hackel, B. J. & Sarkar, C. A. *Quantitative fundamentals of molecular and cellular bioengineering*. (Mit Press, 2020).
8. Johnson, M. L. Why, when, and how biochemists should use least squares. *Anal. Biochem.* **206**, 215–225 (1992).
