# Supplementary material for: An integrated technology for quantitative wide mutational scanning of human antibody Fab libraries
Source: Nat Commun. 2024 May 10;15:3974. doi: 10.1038/s41467-024-48072-z (PMC11087541; doi:10.1038/s41467-024-48072-z)
Supplement: Supplementary file 2 — Description of Additional Supplementary Files [file 41467_2024_48072_MOESM2_ESM.pdf]

<u>Description of Additional Supplementary Files</u>

Supplementary Data 1 A complete list of plasmids, libraries, gene blocks, and primers

Supplementary Data 2 4A8 titration data

Supplementary Data 3 LASSO regression for all variants

Supplementary Data 4 Processed antibody sequence-binding datasets
