# Supplementary material for: An integrated technology for quantitative wide mutational scanning of human antibody Fab libraries
Source: Nat Commun. 2024 May 10;15:3974. doi: 10.1038/s41467-024-48072-z (PMC11087541; doi:10.1038/s41467-024-48072-z)
Supplement: Supplementary file 7 — Reporting Summary [file 41467_2024_48072_MOESM7_ESM.pdf]

nature portfolio

Corresponding author(s): Whitehead TA

Last updated by author(s): Feb 28 2024

Reporting Summary

Nature Portfolio wishes to improve the reproducibility of the work that we publish. This form provides structure for consistency and transparency in reporting. For further information on Nature Portfolio policies, see our Editorial Policies and the Editorial Policy Checklist.

Statistics

For all statistical analyses, confirm that the following items are present in the figure legend, table legend, main text, or Methods section.

n/a	Confirmed	
☐	☒	The exact sample size (*n*) for each experimental group/condition, given as a discrete number and unit of measurement
☐	☒	A statement on whether measurements were taken from distinct samples or whether the same sample was measured repeatedly
☐	☒	The statistical test(s) used AND whether they are one- or two-sided<br>*Only common tests should be described solely by name; describe more complex techniques in the Methods section.*
☒	☐	A description of all covariates tested
☒	☐	A description of any assumptions or corrections, such as tests of normality and adjustment for multiple comparisons
☐	☒	A full description of the statistical parameters including central tendency (e.g. means) or other basic estimates (e.g. regression coefficient) AND variation (e.g. standard deviation) or associated estimates of uncertainty (e.g. confidence intervals)
☐	☒	For null hypothesis testing, the test statistic (e.g. *F*, *t*, *r*) with confidence intervals, effect sizes, degrees of freedom and *P* value noted<br>*Give P values as exact values whenever suitable.*
☒	☐	For Bayesian analysis, information on the choice of priors and Markov chain Monte Carlo settings
☒	☐	For hierarchical and complex designs, identification of the appropriate level for tests and full reporting of outcomes
☐	☒	Estimates of effect sizes (e.g. Cohen's *d*, Pearson's *r*), indicating how they were calculated

Our web collection on statistics for biologists contains articles on many of the points above.

Software and code

Policy information about availability of computer code

Data collection	Data was collected using a Sony SH800 cell sorter and stored as .fcs files. Additional deep sequencing daya was collected using Illumina sequencers as stated in methods at Rush University sequencing core and stored as .fastq files.
Data analysis	All custom scripts and code are freely available on GitHub (https://github.com/WhiteheadGroup/MAGMA-seq). Clonal analyses were performed using VGenes (https://wilsonimmunologylab.github.io/VGenes/).

For manuscripts utilizing custom algorithms or software that are central to the research but not yet described in published literature, software must be made available to editors and reviewers. We strongly encourage code deposition in a community repository (e.g. GitHub). See the Nature Portfolio guidelines for submitting code & software for further information.

Data

Policy information about availability of data

All manuscripts must include a data availability statement. This statement should provide the following information, where applicable:

- Accession codes, unique identifiers, or web links for publicly available datasets
- A description of any restrictions on data availability
- For clinical datasets or third party data, please ensure that the statement adheres to our policy

Processed deep sequencing data is available on sequencing read archive (SRA Deposition #: SRR26328231 - SRR26328333). The plasmids for constructing

compatible workflow Fabs pBDP, pMMP_kappa, pMMP_lambda, pYSD_kappa_mRFP, and pYSD_lambda_mRFP, as well as positive control plasmids p4A8_S7T_BC and p4A8_M59I_T94M_BC, are freely available from AddGene (Deposit: 84029)

Research involving human participants, their data, or biological material

Policy information about studies with human participants or human data. See also policy information about sex, gender (identity/presentation), and sexual orientation and race, ethnicity and racism.

Reporting on sex and gender	N/A
Reporting on race, ethnicity, or other socially relevant groupings	N/A
Population characteristics	N/A
Recruitment	N/A
Ethics oversight	N/A

Note that full information on the approval of the study protocol must also be provided in the manuscript.

Field-specific reporting

Please select the one below that is the best fit for your research. If you are not sure, read the appropriate sections before making your selection.

☒ Life sciences ☐ Behavioural & social sciences ☐ Ecological, evolutionary & environmental sciences

For a reference copy of the document with all sections, see nature.com/documents/nr-reporting-summary-flat.pdf

Life sciences study design

All studies must disclose on these points even when the disclosure is negative.

Sample size	Sample size 'n' was the total number of unique variants observed from libraries.
Data exclusions	No data were excluded from the analysis.
Replication	For the mixed antigen, mixed antibody sort replication was performed using separately prepared yeast libraries, with sorting and sequencing performed on separate days. Replication of the 4A8 sort was performed using isogenic titrations of individual 4A8 sequence variants. The mixed antigen, mixed antibody sort was replicated twice and both attempts were successful.
Randomization	All mutational intermediates were tested and therefore randomization was not necessary.
Blinding	No blinding was necessary as all selections were performed without human intervention.

Reporting for specific materials, systems and methods

We require information from authors about some types of materials, experimental systems and methods used in many studies. Here, indicate whether each material, system or method listed is relevant to your study. If you are not sure if a list item applies to your research, read the appropriate section before selecting a response.

Materials & experimental systems

n/a	Involved in the study	
☐	☒	Antibodies
☐	☒	Eukaryotic cell lines
☒	☐	Palaeontology and archaeology
☒	☐	Animals and other organisms
☒	☐	Clinical data
☒	☐	Dual use research of concern
☒	☐	Plants

Methods

n/a	Involved in the study	
☒	☐	ChIP-seq
☐	☒	Flow cytometry
☒	☐	MRI-based neuroimaging

Antibodies

Antibodies used

We used the following commercial antibodies: anti-V5-AlexaFluor488 (ThermoFisher 37-7500-A488, clone 2F11F7, 1 mg/mL, Lot#:YD372483), Goat anti-hFc-PE (ThermoFisher, 12-4998-82, 0.5 mg/mL, Lot#:2626356).

Validation

These antibodies are sourced from ThermoFisher and are validated by lot at the company and internally in our lab by positive and negative controls of yeast populations. We include controls for yeast display populations not expressing a V5 wpitope tag, and controls with antigens not containing human Fc.
AlexaFluor488 validation:
Elmsaouri S et al. APEX Proximity Labeling of Stress Granule Proteins. Methods Mol Biol. 2022;2428:381-399. doi: 10.1007/978-1-0716-1975-9_23.
Kim EH et al. Development of an HIV reporter virus that identifies latently infected CD4+ T cells. Cell Rep Methods. 2022 Jun 13;2(6):100238. doi: 10.1016/j.crmeth.2022.100238.
Chojnowski A et al. Progerin reduces LAP2α-telomere association in Hutchinson-Gilford progeria. Elife. 2015 Aug 27;4:e07759. doi: 10.7554/eLife.07759.
PE Validation:
Bastard P et al. Influenza-A mediated pre-existing immunity levels to SARS-CoV-2 could predict early COVID-19 outbreak dynamics. iScience. 2023 Nov 14;26(12):108441. doi: 10.1016/j.isci.2023.108441.

Eukaryotic cell lines

Policy information about cell lines and Sex and Gender in Research

Cell line source(s)

Human embryonic kidney HEK293T (female, #CRL-11268), Madin Darby canine kidney (MDCK; female, #CCL-34, NBL-2) and human A549 (#CCL-185) cells were purchased and authenticated by the American Type Culture Collection (ATCC). MDCK-SIAT1 cells were generated previously.

Authentication

Cell lines were not authenticated after receiving from suppliers.

Mycoplasma contamination

Cell lines were not tested for mycoplasma.

Commonly misidentified lines
(See ICLAC register)

N/A

Plants

Seed stocks

Report on the source of all seed stocks or other plant material used. If applicable, state the seed stock centre and catalogue number. If plant specimens were collected from the field, describe the collection location, date and sampling procedures.

Novel plant genotypes

Describe the methods by which all novel plant genotypes were produced. This includes those generated by transgenic approaches, gene editing, chemical/radiation-based mutagenesis and hybridization. For transgenic lines, describe the transformation method, the number of independent lines analyzed and the generation upon which experiments were performed. For gene-edited lines, describe the editor used, the endogenous sequence targeted for editing, the targeting guide RNA sequence (if applicable) and how the editor was applied.

Authentication

Describe any authentication procedures for each seed stock used or novel genotype generated. Describe any experiments used to assess the effect of a mutation and, where applicable, how potential secondary effects (e.g. second site T-DNA insertions, mosiacism, off-target gene editing) were examined.

Flow Cytometry

Plots

Confirm that:

- [x] The axis labels state the marker and fluorochrome used (e.g. CD4-FITC).
- [x] The axis scales are clearly visible. Include numbers along axes only for bottom left plot of group (a 'group' is an analysis of identical markers).
- [x] All plots are contour plots with outliers or pseudocolor plots.
- [x] A numerical value for number of cells or percentage (with statistics) is provided.

Methodology

Sample preparation

S. cerevisiae EBY100 cells are prepared by growth and induction in galactose-containing media. Cells are pelleted and resuspended two times in sterile filtered PBS buffer containing 1 mg/mL BSA.

Instrument

Sony SH800

Software

FloJo is used to process all .fcs files.

Cell population abundance	We sorted at least 200k cells in all post-sort fractions. The exact numbers are given in the processed deep sequencing files.
Gating strategy	Gating strategies for the mixed antibody sorts are provided in Supporting Figures 6 and 9. Briefly, call are FCS-A/SSC-A gated for yeast cells, FSC-H/FSC-A gated for single cells, FSC-H/AF488 gated for displaying cells, and PE gated at different thresholds.

☒ Tick this box to confirm that a figure exemplifying the gating strategy is provided in the Supplementary Information.
